# A review of serological tests available in Brazil for intestinal schistosomiasis diagnosis

**DOI:** 10.1590/0074-02760220236

**Published:** 2023-03-10

**Authors:** Lidia Mara da Silva Ramos, Rosiane A da Silva-Pereira, Edward Oliveira, Cristina Toscano Fonseca, Carlos Graeff-Teixeira

**Affiliations:** 1 Universidade Federal do Espírito Santo, Centro de Ciências da Saúde, Núcleo de Doenças Infecciosas, Vitória, ES, Brasil; 2 Fundação Oswaldo Cruz-Fiocruz, Instituto René Rachou, Grupo de Pesquisa em Biologia e Imunologia de Doenças Infecciosas e Parasitárias, Belo Horizonte, MG, Brasil; 3 Fundação Oswaldo Cruz-Fiocruz, Instituto René Rachou, Grupo de Genômica Funcional de Parasitos, Belo Horizonte, MG, Brasil

**Keywords:** schistosomiasis, serology, diagnosis, Brazil

## Abstract

The World Health Organization (WHO) roadmap and recommendations for elimination of schistosomiasis were recently updated. With significant reductions in the prevalence and intensity of schistosomiasis infections worldwide, there is a need for more sensitive diagnostic methods. There are a few remaining transmission hotspots in Brazil, although low endemicity settings comprise most of the endemic localities. For the latter, serology may represent a tool for population screening which could help eliminate transmission of schistosomiasis. Here, we review serology tests currently available in Brazil from both public health and private laboratories: immunofluorescent antibody tests (IFATs) on adult worm sections and enzyme-linked immunosorbent assays (ELISAs) with soluble egg and adult worm antigens. Both in-house and commercially available tests have received less than adequate performance evaluations. Our review of immediate basic and operational research goals may help identify local adjustments that can be made to improve control interventions aimed at elimination of schistosomiasis as a public health problem.

Schistosomiasis is one of the most important neglected tropical diseases worldwide. Currently, there are approximately 700 million people at risk for infection on several continents.^1^ Sub-Saharan Africa is particularly affected, and there are hot spots of transmission that remain in Brazil, the country with the largest infected population in South America.^2^ A World Health Organization (WHO) roadmap for elimination of schistosomiasis was updated recently, including its published guidelines.^1^ There have been several indicators that a reduction in both the prevalence and intensity of infections has occurred. For example, control measures have produced significant reductions in the prevalence and intensity of infections in many countries.^2^ However, this success has led to new challenges. One is the limited sensitivity of the widely employed Kato-Katz (KK) thick smear.^3^ ^,^ ^4^ Cathodic circulating antigen detection in urine has been extensively used in Africa as a promising more sensitive alternative to parasitological diagnosis, but quality assurance from manufacturers and more extensive specificity evaluation is required, especially for application in low endemicity areas.^5^ ^,^ ^6^ In addition to the need for more sensitive and specific diagnostic tools, there is also a general tendency among physicians and public health personnel to discredit parasitological techniques and to over-value isolated results from molecular and immunological methods. Compared to KK and other parasitological techniques, serological testing is generally considered a more sensitive method.^7^

In Brazil, only egg-positive individuals receive free treatment (praziquantel) under the Unified Health System, Sistema Único de Saúde (SUS).^8^ ^,^ ^9^ However, in recent years, there has been a growing debate regarding the need to expand the criteria used for prescribing praziquantel to include results from serological diagnosis tests. The goal of this review was to examine available serological tests in Brazil to provide insight into the effectiveness of the diagnostic/treatment decision protocol currently employed in Brazil for cases of schistosomiasis. We additionally review data regarding tests that are commercially available worldwide. Most of the tests used to detect schistosomiasis are produced in Germany, France, and Switzerland, as reported by the Global Schistosomiasis Alliance.^10^

Serology testing performed at public health laboratories

In February 2019, a survey of public health laboratories in Brazil disclosed that two collaborating centres offer serology testing for schistosomiasis (Table I). This information was updated and confirmed between May and June 2022. The in-house tests performed include IgM-immunofluorescence antibody tests (IgM-IFAT) (which use male adult worm paraffin sections) and enzyme-linked immunosorbent assays (ELISAs) using soluble adult worm antigen (SWAP) or soluble egg antigen (SEA). Indicators of performance are presented in Table I for IFAT conducted at the Instituto Adolfo Lutz (IAL) and SWAP/SEA ELISAs performed at FIOCRUZ-Minas (IRR). IFATs’ performance indicators have been reported in several studies (Table II).

**TABLE I:** Reference laboratories for schistosomiasis serological tests among the network of public health laboratories in Brazil (Coordenação Geral de Laboratórios de Saúde Publica, CGLAB, Brazilian Ministry of Health), updated May-June 2022

Laboratory acronym	Full title of laboratory	Test performed	Se %	Sp %
IAL	Instituto Adolfo Lutz - São Paulo, Núcleo de Enteroparasitas, do Centro de Parasitologia e Micologia^*a*^	AWA-IgM-IFAT In-house	60-97^*^	84-100^*^
IRR-FIOCRUZ	Instituto René Rachou, FIOCRUZ-MG - Belo Horizonte, Laboratório de Referência em esquistossomose^*b*^	SWAP- IgG ELISA In-house	90^**^	90^**^
		SEA- IgG-ELISA In-house	85^**^	80^**^

*a*: Inst Adolfo Lutz, phone +55 11 3068 2888, Av Dr Arnaldo, 351, 8th floor, CEP 01246-000 - São Paulo; *b*: Inst René Rachou, phone +55 31 3349 7700; Avenida Augusto de Lima 1715, CEP 30190-002 Belo Horizonte MG. Se: sensitivity; Sp: specificity; AWA-IgM IFAT: male adult worm sections, immunofluorescence antibody test; *: see Table III for descriptions of the ranges of performance indicators; **: reference #^11^;SWAP-ELISA: soluble worm antigen, enzyme-linked immunosorbent assay; SEA: soluble egg antigen.

**TABLE II:** Studies that determined performance indicators (sensitivity, specificity) for IgM indirect immunofluorescence tests using paraffin sections of male adult worm as antigen

Reference	Sample	Reference method	Se %	Sp %
^12^	Serum	Egg-detection (KK and HPJ)	94	95
^13^	Serum	CPT	78	87
^14^	Serum	Egg-detection (KK and HPJ)	60	84
^15^	Blood on filter paper	Egg-detected (KK and HPJ)	97	98
^16^	Serum	Egg-detected (KK and MHT)	95	100

Se: sensitivity; Sp: specificity; KK: Kato Katz method; HPJ: Hoffman, Pons and Janer (Lutz) method, spontaneous sedimentation; CPT: Circumoval Precipitin Test; MHT: miracidium hatching test.

Serology testing performed at private laboratories

In 2009, Brazil had 16,657 clinical laboratories.^17^ Most of them did not support testing for schistosomiasis and they forwarded their samples to clinical laboratories that could perform the tests. Among the support laboratories in Brazil are Diagnósticos da América SA (DASA), Hermes Pardini, Diagnósticos do Brasil, and Fleury. Together, these support laboratories represent more than 30% of the market share in Brazil, and all four provide serological testing for schistosomiasis (Table III).^18^ The diagnostic kits that are produced by Euroimmun and NovaTec (Germany) constitute nearly all the serological tests performed by private laboratories in Brazil. For one of the support laboratories (Fleury), their samples are shipped to a Mayo Clinic in the United States where testing is provided with the SEA-ELISA-IgG NovaTec kit. Unconfirmed information indicates that the NovaTec kit was validated at the Mayo Clinic using another serological test as a reference method: a fluorescent microsphere immunoassay (Focus Labs / QuestDiagnostics, USA; https://testdirectory.questdiagnostics.com/test/home), utilising the crude microsomal fraction of adult *Schistosoma mansoni* worms (MAMA).^19^ This less than adequate accuracy evaluation will be addressed below (see “performance indicators”).

**TABLE III:** Clinical analysis support laboratories providing serological testing for schistosomiasis in Brazil. Updated June 2022

Laboratory acronym	Full title of laboratory	Website	Test and supplier
DB	DB Diagnósticos do Brasil	https://www.diagnosticosdobrasil.com.br/	SEA-ELISA-IgG NovaTec, Germany
PARDINI	Hermes Pardini	https://www.hermespardini.com.br/	AWA-IFAT-IgG Euroimmun, Germany
DASA	DASA	https://dasa.com.br/	AWA-ELISA and IFAT Euroimmun, Germany
FLEURY	Fleury Medicina e Saúde	https://www.fleury.com.br/	SEA-ELISA-IgG NovaTec, Germany

SEA: soluble egg antigen; ELISA-IgG: enzyme-linked immunosorbent assay to detect G class antibodies; AWA: adult worm antigen; IFAT: indirect immunofluorescent antibody test.

Both advice received from several workers in well-known Brazilian private laboratories, as well as results from an Internet search conducted in March 2022, indicate that the main support laboratories are DB, PARDINI, DASA, and FLEURY (Table III). This information is also in accordance with a report by SEBRAE.^18^

Private company data

It was particularly difficult to obtain detailed information from private laboratories and commercial sources regarding diagnostic kits. “Commercial secrecy” was cited as a factor in some cases. In addition, fact sheets from commercially available products sometimes lacked essential data or had versions that provided different information. For example, data sheets provided by Euroimmun, “FI _2300G_ BR_C06.doc Version: 11/01/2017”, entitled “IF: *Schistosoma mansoni* IgG” (File name beginning with “FI_2300G_IF”), as well as a data sheet with the same reference, “FI _2300G_ BR_C06.doc, Version: 11/01/2017”, yet entitled, “IF: *Schistosoma mansoni* IgM” (File name beginning with “FI_2300M_IF”), both presented performance indicators for IFAT-IgG and IFAT-IgM, yet with minor differences. File “FI_2300M_IF”, and not file “FI_2300G_IF”, presents data regarding cross-reacting sera from individuals infected with *Echinococcus granulosus*. However, the very small number of samples tested (13 with IFAT-IgM and 20 with IFAT-IgG) prevents any useful accuracy estimations (lines 4 and 7, Table IV). Furthermore, a more robust evaluation of specificity with an adequate number of sera from individuals with other parasitic infections is lacking for IFATs. Samples from “German reference centres” (14 samples) were used to estimate a specificity of 100%, which is also inadequate (line 3, Table IV).

**TABLE IV:** Performance indicators of commercially available indirect immunofluorescence tests (IFATs) for schistosomiasis diagnosis in Brazil, June 2022

Source	System	References	Se %	Sp %	Line
Euroimmun (#1)	Male worm IgG Frozen	Egg-positive; 46 samples	95		1
		German “healthy” blood donors; 200 samples		99	2
		German reference centres; 14 samples		100	3
		*Echinococcus granulosus* infected individuals; 20 samples		62	4
Euroimmun (#2)	Male worm IgM Frozen	Egg-positive; 46 samples	91		5
		German “healthy” blood donors; 200 samples		98	6
		*Echinococcus granulosus* infected individuals; 13 samples		62	7

Se: sensitivity; Sp: specificity. According to the technical information available from suppliers: (#1) FI _2300G_ BR_C06.doc, Version: 11/01/2017”, but entitled, “IF: Schistosoma mansoni IgG” (File name beginning with “FI_2300**G**_IF”), and (#2) FI _2300G_ BR_C06.doc, Version: 11/01/2017 (File name beginning with “FI_2300**M**_IF”), are both provided by Euroimmun Brasil Medicina Diagnóstica Ltda, on behalf of Euroimmun-PerkinElmer Company, Germany.

Another commercial source for diagnostic kits in Brazil is NovaTec. The fact sheet we had access to (Table V) does not provide a reference method for defining true-positive samples and states only that the plates are covered with “specific antigen”. A description of the antigen was only obtained after consulting the technical support department of NovaTec.

**TABLE V:** Performance indicators of commercially available ELISAs for schistosomiasis diagnosis in Brazil, June 2022

Source	System	References	Se % (95% CI)	Sp % (95% CI)	Line
Euroimmun (#1)	SEA-ELISA IgG	ELISA “A” 118 samples	73	98	1
		ELISA “B” 117 samples	93	79	2
		IFAT IgG Euroimmun; 593 samples	77	90	3
		Other parasites; 114 samples		92	4
		Other conditions (rheumatoid & viral infections); 96 samples		98	5
		
		“Healthy” children (< 3y); 25 samples		100	6
		“Healthy” children (3-10y); 63 samples		98	7
		Pregnant women; 100 samples		92	8
		“Healthy” blood donors; 500 samples		97	9
Euroimmun (#2)	AWA-ELISA IgM	ELISA “C” (*); 90 samples	98	97	10
		Other parasites; 101 samples		80	11
		Virus, bacteria, autoantibodies; 169 samples		87	12
NovaTec (#3)	SEA-ELISA IgG	Not described	93 (82.8-98.7)	98 (92.2-99.9)	13
NovaTec (#4)	AWA-ELISA IgM	Not described	92	96	14

SEA: soluble egg antigen; ELISA: enzyme-linked immunosorbent assays; ELISA Euroimmun results were compared to samples characterised by ELISA from other, yet undisclosed, sources; IFAT: immunofluorescent antibody test; AWA: adult worm antigen. According to the technical information available: (#1) EI_2300-9601G_C02, Version: 25/July/2018 and (#2) EI_2300-9601_C01, Version: 23/March/2018 are both provided by Euroimmun Brasil Medicina Diagnóstica Ltda, on behalf of Euroimmun-PerkinElmer Company, Germany. (#3) SCHG0410_2020-07-14_Ka-ab Lot 068 and (#4) SCHM0410_2020-10-12_Ka-ab Lot 007 are both provided by NovaTec Immundiagnostica GmbH, 63128 Dietzenbach, Germany, June 2022. CI: confidence interval.

Costs of serology for schistosomiasis

Between March and June 2022, we investigated the costs of IFAT and ELISA kits. Costs were provided by six private laboratories. The average final cost per sample for consumers was estimated at US$ 30.69 (13.9 - 48.18) for IFAT and US$ 4.88 (2.88-5.87) for ELISA (US$ 1 = R$ 5.22, 22 June 2022). The kits were produced by Euroimmun and NovaTec, respectively.

Other diagnostic kits available from Germany, France, and Switzerland are presented in Table VI. The “Free On Board” (FOB) costs per sample (without considering importation expenses) varied from US$ 10.07 to US$ 16.48 for ELISA (average price, US$ 13.31). There is one immunochromatographic test that cost US$ 20.42 and an immunoagglutination assay that cost US$ 7.76 (US$ 1 = R$ 5.22) as of 22 June 2022.

**TABLE VI:** Commercially available diagnostic kits for schistosomiasis (other than Euroimmun and NovaTec, included in Tables IV and V). Modified from GSA.^(10)^ Costs per sample updated in June 2022 (US$ 1 = R$ 5.22)

Commercial source	Method	Antigen	Se %	Sp %	Cost per sample (US$)	Representative in Brazil	Line
Bordier Switzerland	ELISA-IgG	SEA & SWAP	94	99	12.72	No	1
DRG Germany	ELISA- IgG (3512)	SEA	85	100	10.07	Yes	2
	ELISA IgG (3872)	SEA	93	98	16.48		3
	ELISA IgM (5904)	AWA	92	96	15.41		4
LDBio France	ICT IgG	AWA	95	92	20.42	No	5
	ICT IgM	AWA	Ni	Ni			6
ELITECH France	IHA	AWA	85	98	07.76	Yes	7
TECAN Switzerland	ELISA IgG	SEA	93	98	11.87	Yes	8
	ELISA IgM	SEA	Ni	Ni	Ni		9
VIRAMED Germany	ELISA IgG (3872) From DRG	SEA	93	98	16.48	Yes	10
IVD USA	ELISA IgG (3512) From DRG	SEA	85	100	10.07	Yes	11
BIOSYNEX France	IHA	Ni	Ni	Ni	Ni	Ni	12

Se: sensitivity; Sp: specificity; ELISA: enzyme-linked immunosorbent assay; SEA: soluble egg antigen; SWAP: soluble worm antigen; ICT: immunochromatographic test; AWA: adult worm antigen; Ni: not informed; IHA: indirect hemagglutination assay.

Performance indicators

For validation of diagnosis tests, both sensitivity (percentage of positive results among true positive samples) and specificity (percentage of negative results among true negative samples) are important performance indicators.^20^ These indicators are influenced by the reference test used to determine the status of infection as true positive and true negative samples. In cases of schistosomiasis, egg detection is direct evidence of an active infection, and the best definition for true-positive samples to be used as reference standards for evaluating indirect methods, such as serology. However, unless faecal samples are collected on different days and are extensively examined, an infection cannot be ruled out among individuals living in endemic areas due to the detection limit of most parasitological tests which excludes detection of low intensity infections. Egg detection was used as the reference in four out of the five studies that previously reported sensitivity and specificity data for IFAT performed at IAL (Table II), and in evaluations reported in fact sheets for Euroimmun’s IFAT (Table IV). However, neither egg detection was used to evaluate ELISA kits from any supplier, nor was the reference method disclosed in the NovaTec data sheet (Table V). A comparison of one serological test using another serological test as reference is not an appropriate test design for accuracy determination, especially when the reference serology is not disclosed (Table V; lines 1,2,3,10), or when the number of reference sera is omitted (Table V; lines 13 and 14).

Euroimmun data sheets report testing of specificity control sera for evaluations of ELISAs (Table V). The sera tested included samples from patients with other infections/conditions and large numbers of “healthy” blood donors. However, specificity estimates from small sample sets are not reliable (see Table V, line 6; Table IV, lines 3,4,7). It is also not adequate to state that 100% IFAT specificity is estimated when only 14 serum samples were tested (Table IV, line 3).

Discussion and conclusions

The results of this review highlight the need for careful and well-controlled studies to be conducted for further evaluations of diagnostic tests. The results also confirm many of the recommendations published in a recent review of SEA-ELISAs.^21^ Two broad applications for serology are: (i) clinical investigations of individuals, and (ii) population-based studies to screen for prevalence. In non-endemic areas, the former application predominates. Interpretation of serological tests in clinical settings like neuroschistosomiasis and liver transplantation is greatly impaired by lack of appropriate performance evaluations. Non-endemic countries produce all the commercial serology tests available to date. Meanwhile, use of serology for investigating patients suspected of schistosomiasis in endemic areas is highly problematic since some studies have reported persistence of antibodies for some months after treatment, which could preclude the correct discrimination of active infections, as well as a clear demonstration of schistosomiasis as the etiologic agent.^22^ ^,^ ^23^ Therefore, especially in areas trying to reach low levels of endemicity, a two-step procedure involving a highly sensitive test followed by a highly specific test is a very promising strategy. This two-step approach is recommended by the WHO.^1^

At the individual level, there has been an urgency and tendency for both physicians and patients to overemphasise isolated laboratory results to quickly reach a conclusion regarding an etiological diagnosis. Ideally, health personnel should be motivated to gather as much information as possible for a differential diagnosis to ensure the safe management of patients. In Brazil, optimisation and updating of diagnostic workflows are urgent tasks.

For any application, serological tests must be adequately standardised and evaluated. Initially, evaluations are performed under optimal conditions in a laboratory to characterise “efficacy”. Performance evaluations of antibody-detection methods should avoid comparison with another test of the same category. A consolidated standard reference should combine sensitive egg-, DNA- or antigen-detection methods. Subsequently, evaluations are conducted under field conditions to determine “effectiveness” (validation). The latter is especially relevant for rapid diagnostic tests developed for point-of-care applications.^20^

It is noteworthy that very complex antigens, like SEA and AWA, have labor-intensive procedures for their preparation. Unfortunately, this can also lead to poor inter-batch reproducibility. To improve serology tests, use of well-characterised recombinant antigens is needed. Biobanks of well-characterised reference samples would be extremely useful for test evaluations performed with joint coordination between the WHO, governments, and other organisations, such as the Foundation for Innovative New Diagnostics (FIND) or Oswaldo Cruz Foundation (FIOCRUZ-Brazil). Researchers should also follow recommendations for reporting essential information needed in data sheets of commercially supplied diagnostic kits, like what has been formulated by the initiative, “Standards for the Reporting of Diagnostic accuracy” (STARD-2015).^24^

Complexity of antibody detection systems, like immunofluorescent testing on worm sections (IFAT), is also an obstacle for wide use at populational screening, while ELISA is clearly advantageous for automation and large-scale applications. More valuable yet are point-of-care rapid diagnostic methods attending to ASSURED criteria for effective implementation in field conditions.^25^

As indicated in the updated guidelines of the WHO, diagnosis is a key element for elimination of schistosomiasis.^1^ It has been demonstrated that serology is a valuable screening tool for infections in communities. For schistosomiasis, serology would be especially helpful in guiding the late stages of control efforts to achieve interruption of transmission. Serology could also serve as a certification criterion and provide post-elimination surveillance. Concomitantly, best practices for reagent production, quality assurance, and adequate evaluation of performance are essential steps of an authorisation process at the national level (*e.g.*, in Brazil, ANVISA).
